# Non-Invasive Estimation of Machining Parameters during End-Milling Operations Based on Acoustic Emission

**DOI:** 10.3390/s20185326

**Published:** 2020-09-17

**Authors:** Andrés Sio-Sever, Erardo Leal-Muñoz, Juan Manuel Lopez-Navarro, Ricardo Alzugaray-Franz, Antonio Vizan-Idoipe, Guillermo de Arcas-Castro

**Affiliations:** 1 Grupo de Investigación en Instrumentación y Acústica Aplicada, Departamento de Ingeniería Mecánica, Universidad Politécnica de Madrid, 28040 Madrid, Spain; 2 Departamento de Ingeniería Mecánica, Universidad de La Frontera, Temuco 4780000, Chile; erardo.leal@ufrontera.cl (E.L.-M.); ricardo.alzugaray@ufrontera.cl (R.A.-F.); 3 Grupo de Investigación en Instrumentación y Acústica Aplicada, Departamento de Telemática y Electrónica, Universidad Politécnica de Madrid, 28040 Madrid, Spain; juanmanuel.lopez@upm.es; 4 Departamento de Ingeniería Mecánica, Universidad Politécnica de Madrid, 28006 Madrid, Spain; antonio.vizan@upm.es

**Keywords:** microphone, MEMS, machining, milling, acoustic emission, process monitoring

## Abstract

This work presents a non-invasive and low-cost alternative to traditional methods for measuring the performance of machining processes directly on existing machine tools. A prototype measuring system has been developed based on non-contact microphones, a custom designed signal conditioning board and signal processing techniques that take advantage of the underlying physics of the machining process. Experiments have been conducted to estimate the depth of cut during end-milling process by means of the measurement of the acoustic emission energy generated during operation. Moreover, the predicted values have been compared with well established methods based on cutting forces measured by dynamometers.

## 1. Introduction

The improvement of industrial processes demands new technologies that provide accurate information regarding the stationary or dynamic conditions of their operations. In the case of machining processes, the knowledge of operating conditions is required towards efficiency improvement and quality optimization.

A wide range of methods have been developed to monitor milling processes, most of them are based on the measurement of the cutting forces present in the workpiece because it represents accurately most of common machining phenomena. Cutting force methods have the advantage of providing accurate results [1,2,3] and they are simple to implement, but they have the disadvantage of requiring high cost transducers and direct contact between the sensor and the workpiece.

Other detecting methods rely on ultrasound emission and reception to measure the depth of cut in milling applications [4], thus solving the direct contact requirement. More recently, the contact between the cutting tool and the workpiece has been studied with their electrostatic contact to determine the engagement of each tooth [5,6].

Acoustic emission (AE) techniques have received increasing attention during the last few years due to the ability to perform multiple monitoring tasks during machining processes. One of the first applications was the detection and study of tool and workpiece contact in machining [7,8], which quickly led to an interest in the automatic detection of malfunctions. In the event of a tool with broken or blunt teeth, acoustic emission technologies can automatically detect their presence with the use of relatively simple setups [9,10,11,12,13]. Even if the tool is in good working condition, phenomena like runout [14,15] or chatter [16,17,18,19] are common problems that modern industry has to face, but due to the change in the acoustic emission generated by each of those phenomena they can be readily detected and solved.

Beyond their conventional uses in traditional machining, the continuous research on AE allowed it to have newfound popularity in more subtle applications, such as roughness analysis [20] and the study of micromachining processes [21]. The main advantages of using AE sensors rely on their low invasiveness and the possibility to assess different sources of interest during the machining process.

### Acoustic Emission

Acoustic emission has attained notable focus in the engineering community in recent years, both in contact sensors [22,23,24,25] and non-contact ones [26,27,28] in a wide range of applications. Besides the use of AE sensors in the machining field, it has also been used in detecting weak points in metallic structures such as piping [29], aircraft [30], windmills [31] and even bone-milling surgery [32].

Acoustic emission refers to the mechanical waves generated by the plastic (irreversible) changes in solid bodies during the application of stresses that can produce cracks, deformations and movement of dislocations among others, as well as friction and other phenomena [33].

Traditionally, AE is obtained by means of piezoelectric transducers that are directly attached to the surface of the specimen under load [22,23,24,25], which requires the use of expensive sensors that may obstruct the pathway of the cutting tool and whose proximity to the cutting fluid may be dangerous to the integrity of the sensing device. To solve the intrusiveness and cost issues, the use of microelectromechanical systems (MEMSs) was chosen in the present work.

MEMS technology was initially applied to microelectronics because of the small form factor required by the ever increasing processing needs of computers. Due to the elevated cost of such devices, their mass implementation outside the field of computation was hardly profitable, but the recent developments in MEMS manufacturing [34,35,36,37,38,39,40,41,42] suggest that they can potentially be used in numerous applications where their small size and cost reduction [43,44,45,46,47] can offer an unobtrusive and affordable solution.

In machining processes, AE might be one of the best options to analyze subsurface damage and anisotropic materials [48] because of the capabilities of this technology of detecting weak points and discontinuities inside solid bodies without the need to perform any destructive testing.

These monitoring capabilities are not limited to milling, and with some modifications, it can also be applied to operations such as drilling [49], boring or turning [50], as all of them would benefit from tool monitoring [51].

The measurement of airborne AE signals poses a big challenge in filtering the background noise, which is produced by the machine under study and by other industrial noise sources present in the surrounding environment. Previous works have shown different approaches to find a flexible method that works under different operating conditions without compromising accuracy. For example, the use of neural networks by Gaja et al. [52], fuzzy logic algorithms [53,54,55], and radial basis functions [56,57] have proven to be effective methods to estimate the axial depth of cut based on AE.

With the aforementioned state of the art in mind, the objective of the present work is to analyze the performance and applicability of a truly non-invasive and low-cost alternative AE measurement system with the ability to estimate the cutting conditions during end-milling operations. In order to do so, a system prototype has been developed and tested in real operating conditions, and the accuracy of the method has been compared with the results of well established methods based on the measurement of the cutting forces [3].

Section 1 describes the context of this work and provides the theoretical background. Section 2 includes the geometrical model used to characterize the signal pattern of the cutting forces in end-milling processes and Section 3 expands on its application in system that is being studied. Section 4 presents the materials and methods used in the development of a measuring system prototype, including the design of the signal conditioning unit, the hardware and software architecture of the system, the calibration and signal processing algorithms. Section 5 presents and discusses the experimental results from both a qualitative and a quantitative point of view. Finally, Section 6 summarizes the main findings this work.

## 2. Geometrical Model of the End-Milling Process

**Figure 1:**
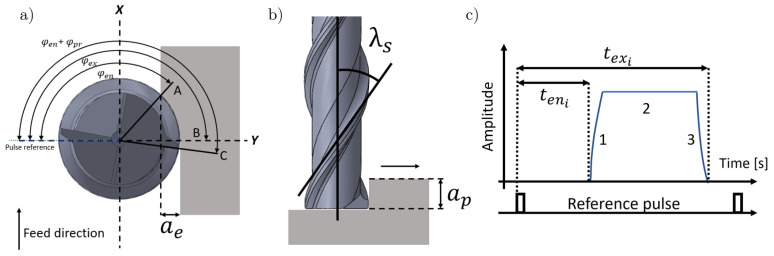
Main parameters of the end-milling process. (**a**) Top view, (**b**) front view, and (**c**) time history of the cutting process.

The machining conditions in end-milling process can be obtained in terms of the geometrical characteristics of the cutting tool. This means, that the radial depth of cut (a_e_) and the axial depth of cut (a_p_) can be related to the characteristic angles of the tool flute (entry angle $\varphi_{en}$, exit angle $\varphi_{ex}$, projected angle $\varphi_{pr}$ and flute angle $\lambda_{s}$) shown in Figure 1a,b.

If the diameter of the tool (D) is known, the relationship between the entry angle of the tool and the width of cut can be established geometrically as shown in Equation (1):

(1) $$\varphi_{en_{i}}=\pi -arcos\left(1-\frac{2a_{e_{i}}}{D}\right)$$

Furthermore, if a fixed geometrical reference is set, and a time base is available, this angle can be determined by measuring the time it takes the flute to complete that part of the rotation. This can be done by adding a digital pulse for each revolution of the spindle as shown in Figure 1c. The associated time parameter is denoted as the entry time (*t_en_i__*) and it represents the time, measured from the reference pulse, that takes the cutting edge of the tool to get in contact with the workpiece on each spindle revolution.

The entry time and entry angle are related by the spindle period (*T*) as shown in Equation (2):

(2) $$t_{en_{i}}=\frac{\varphi_{en_{i}}}{2\pi }T$$

The width of cut can be obtained in terms of the tool diameter (*D*), the entry time (*t_en_i__*) and the spindle period (*T*) (see Equation (3)).

(3) $$a_{e_{i}}=\frac{D}{2}\left(1+cos\left(1-\frac{2\pi t_{en_{i}}}{T}\right)\right)$$

Analogously, the projected time depends on the projected angle of the active flute over the working plane of the workpiece being machined (φ*_pr_*). This angle can be obtained by measuring the time it takes each flute of the tool to travel the projected angle as shown in Equation (4).

(4) $$t_{pr_{i}}=\frac{T}{2\pi }\varphi_{pr_{i}}=\frac{Ta_{p_{i}}tan\lambda_{s}}{\pi D}$$

Therefore, the depth of cut can be obtained in terms of the projected angle (φ*_pr_*) and the spindle period (*T*) of the tool as shown in Equation (5).

(5) $$a_{p_{i}}=\frac{\pi D}{Ttan\lambda_{s}}t_{pr_{i}}$$

## 3. Cutting Force Model

**Figure 2:**
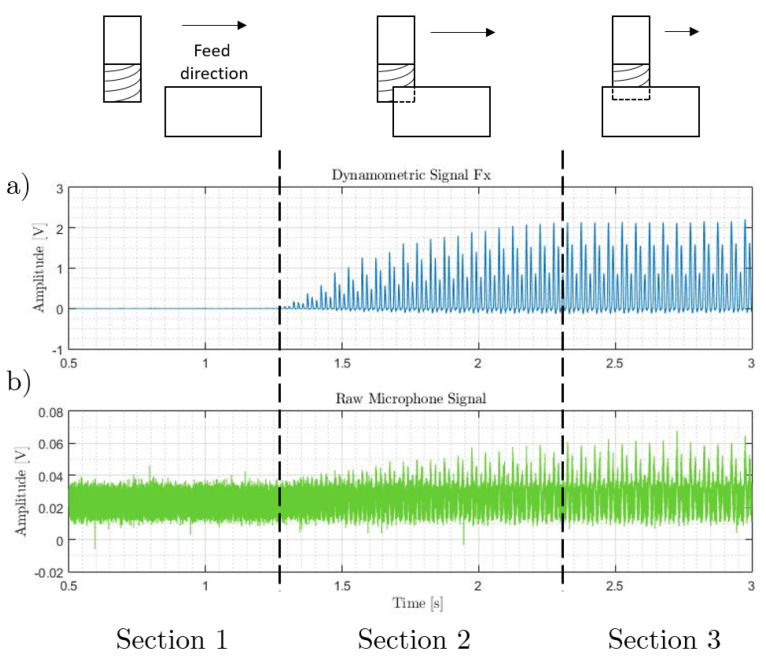
Phases of the end-milling process. (**a**) Cutting force signal, and (**b**) acoustic emission (AE) signal.

One of the main problems in measuring airborne acoustic emissions during machining processes is dealing with background noise. Among the main sources one can find electrical noise, environmental noise, and some other internal noise sources from the machine under analysis. This effect can be noticed in Figure 2, when comparing the experimental signals of the cutting forces measured by a dynamometer and the acoustic emission registered from a non-contact microphone during end-milling operations. In the case of the cutting forces, there are three clearly marked phases depending on the degree of engagement between the cutting tool and the workpiece. In the first phase, the cutting tool is approaching the workpiece with no material removal; the second phase, where the tool is entering into the workpiece; and the third phase, the zone of uniform cut once the tool is fully engaged. These features are not easy to find on the acoustic emission signal, and therefore signal filtering is required. In this work an adaptive filter is used, taking the synthesized model of cutting forces proposed by Leal et al. [3] as the reference signal.

The formulation of the geometric model described in the previous section can be extended to the three cutting phases shown in Figure 1c, then the instantaneous axial depth of cut can be rewritten as shown in Equation (6).

(6) $$a_{pa}\left(\varphi \right)=\left\{\begin{array}{cc} \frac{\varphi_{j}-\varphi_{en}}{\varphi_{pr}}a_{p} & \varphi_{en}\leq \varphi_{j}\leq \varphi_{ex}\\ \frac{\varphi_{ex}-\varphi_{en}}{\varphi_{pr}}a_{p} & \varphi_{ex}\leq \varphi_{j}\leq \varphi_{en}+\varphi_{pr}\\ \frac{\varphi_{ex}-\left(\varphi_{j}-\varphi_{pr}\right)}{\varphi_{pr}}a_{p} & \varphi_{en}+\varphi_{pr}\leq \varphi_{j}\leq \varphi_{ex}+\varphi_{pr} \end{array}\right.$$

Equation (7) allows the determination of the average chip thickness ($\bar{h}$(φ)) on each of the three cutting phases, where *f_z_* is the feed per tooth in mm.

(7) $$\bar{h}\left(\varphi \right)=\left\{\begin{array}{cc} \frac{1}{\varphi_{j}-\varphi_{en}}f_{z}\left[cos\left(\varphi_{en}\right)-cos\left(\varphi_{j}\right)\right] & \varphi_{en}\leq \varphi_{j}\leq \varphi_{ex}\\ \frac{1}{\varphi_{ex}-\varphi_{en}}f_{z}\left[cos\left(\varphi_{en}\right)-cos\left(\varphi_{ex}\right)\right] & \varphi_{ex}\leq \varphi_{j}\leq \varphi_{en}+\varphi_{pr}\\ \frac{1}{\varphi_{ex}-\left(\varphi_{j}-\varphi_{pr}\right)}f_{z}\left[cos\left(\varphi_{j}-\varphi_{pr}\right)-cos\left(\varphi_{ex}\right)\right] & \varphi_{en}+\varphi_{pr}\leq \varphi_{j}\leq \varphi_{ex}+\varphi_{pr} \end{array}\right.$$

**Figure 3:**
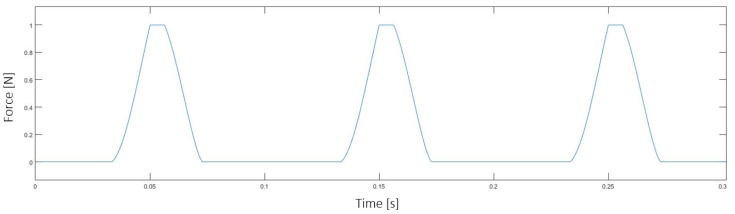
Cutting force model for a single edge cutting tool, with D = 8 mm, a_p_ = 8 mm, a_e_ = 1 mm, f_z_ = 0.08 mm and n = 1200 rpm.

Thus, the three components of the cutting forces can be obtained from Equations (8)–(10):

(8) $$F_{t}\left(\varphi \right)=k_{t}\left(\varphi \right)a_{pa}\bar{h}\left(\varphi \right)$$

(9) $$F_{r}\left(\varphi \right)=k_{r}\left(\varphi \right)a_{pa}\bar{h}\left(\varphi \right)$$

(10) $$F_{a}\left(\varphi \right)=k_{a}\left(\varphi \right)a_{pa}\bar{h}\left(\varphi \right)$$

where *k_t_*, *k_r_* and *k_a_* correspond to the cutting force coefficients for tangential, radial and axial directions, respectively. These coefficients are obtained experimentally and adjusted for each material. This set of equations can be used to model the cutting forces as shown in Figure 3, where the entry and exit points of the cutting edge on each tool revolution are time parameters needed to estimate the depth of cut. This signal is generated from the nominal cutting conditions and the cutting tool geometry, and it represents the reference signal for the adaptive filter described in Section 4.1.

## 4. Materials and Methods

This section describes the design of the prototype measuring system, and the experimental setup used to perform the validation of the method.

### 4.1. Prototype Measuring System

The prototype proposed in this work is a combination of hardware and software to measure the airborne acoustic emission during the end-milling process in order to estimate the depth of cut. The transducer selected for this application is the Knowles SPU1410LR5H-QB. This corresponds to a low-cost and non-contact microelectromechanical systems (MEMS) microphone with the ability to measure high frequency content of acoustic emissions [7,8] but at expense of amplitude precision. The technical specifications of this transducer are listed in Table 1.

**Figure 4:**
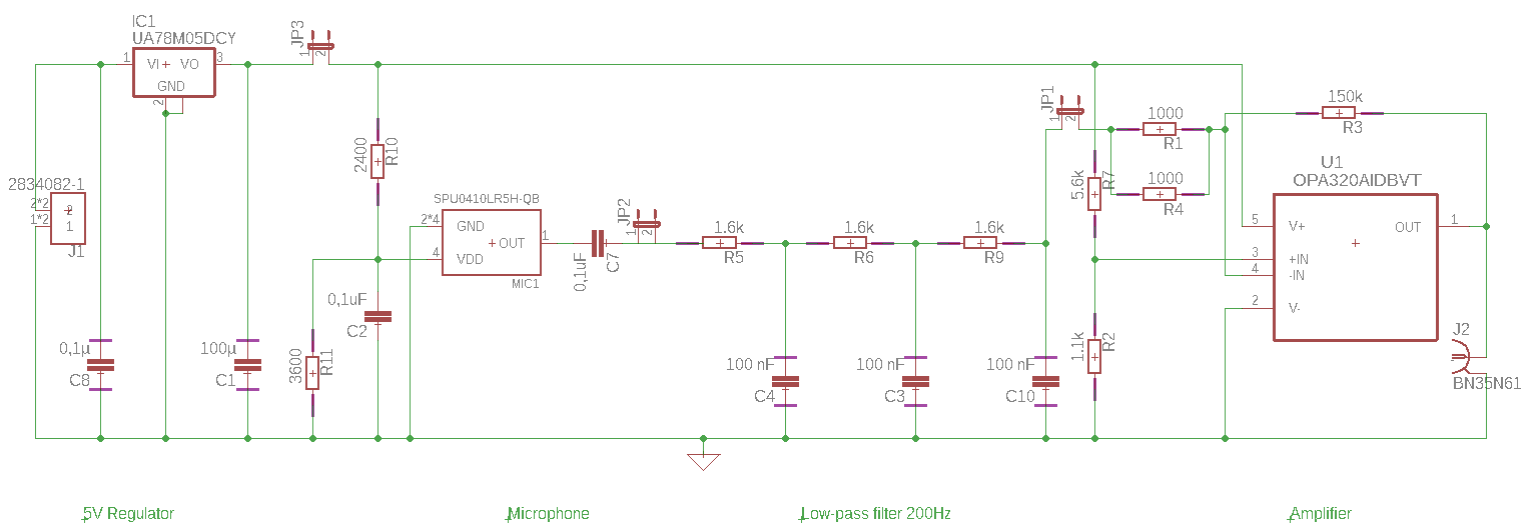
Schematic representation of the conditioning board.

Additionally, a signal conditioning board has been designed to provide power supply to the microphone and also to handle basic amplification and filtering. Its schematic circuit is depicted in Figure 4, where IC2, C1 and C8 provide a 5 V DC stabilized power supply from a 9 V battery connected to J1. The microphone MC1 is powered from a 3.6 V DC using R10 and R11, and C2 has been added to eliminate power fluctuations. The output of the microphone is connected to a three-stage low pass Butterworth filter with a cut-off frequency of 1 kHz represented by R5, C4, R6, C3, R9 and C10. Finally, the signal amplification stage is composed by U1 and its surrounding components (R2 and R3 for the voltage divider, R1 and R4 for the input resistor and R3 for the feedback resistor). The latter deals with the inherent offset value of the microphone and it also maximizes the achievable dynamic range with a unipolar power supply.

**Figure 5:**
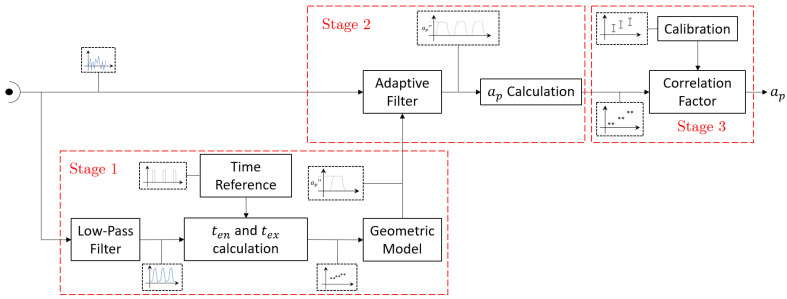
Block diagram of the system.

The analog output from the conditioning board is digitalized by means of a data acquisition system which would be described in the next section. This signal is fed to an algorithm that estimates the axial depth of cut. The block diagram of the main routine is illustrated in Figure 5, where three stages can be identified.

In the first stage, the reference signal for the adaptive filter is generated based on the cutting force model described in Section 3. The second stage, which can be considered as core of the algorithm, applies an adaptive filter to obtain the time parameters needed to compute the depth of cut using Equation (5). Finally, the third stage consists on the estimation of the depth of cut after adjustments that compensate for systematic errors present along the measurement chain.

#### 4.1.1. Stage 1: Generation of Cutting Force Reference Signal

As mentioned previously, the goal of this stage was to provide a reference signal for the adaptive filter on stage 2. This was done by extracting the main features of the acoustic emission signal based on the geometric model of the cutting forces presented in Equations (6)–(10). This strategy was sensitive to changes in the operation conditions, and therefore, this measuring system had the ability to work completely independent of the machining control system.

**Figure 6:**
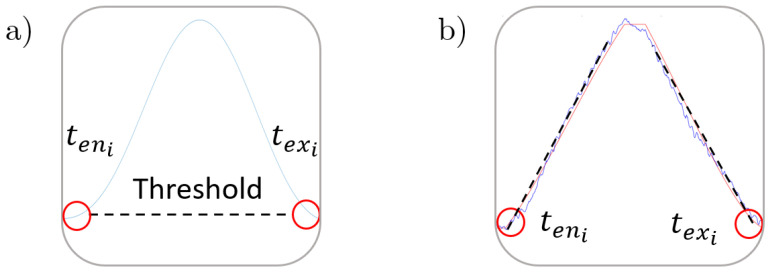
Time parameters measurement. (**a**) Threshold-based algorithm. (**b**) Slope-based algorithm.

Before obtaining the time parameters, a third order Butterworth filter with a cut-off frequency of 150 Hz needed to be applied to the microphone raw signal, in order to remove most of the electrical and background noise. From the low frequency waveform, the entry and exit times were found by detecting the amplitude peaks that exceeded a threshold value (see Figure 6a). This threshold was automatically set from the mean value of the signal while the cutting tool was approaching the workpiece with no material removal. Furthermore, the digital pulse allowed the transformation of time values to the angle domain, which were independent of the spindle speed variations.

#### 4.1.2. Stage 2: Estimation of Depth of Cut Using the Adaptive Filter

At this stage, the AE raw signal was filtered using the reference signal generated in stage 1. Both signals were divided into equally sized segments (windowing), whose size depended on the priorities of the monitoring process. A short window could be used to respond quickly to changes in the process parameters (e.g., width or depth of cut), but it would make the system less robust against background noise, whereas long windows would have the opposite effect. A window width of two tool revolutions (0.1 s) was chosen as a good compromise between these two factors.

The adaptive filter implemented was a 7th order that uses a Normalized Least Mean Squares algorithm (NLMS), which made it stable against any scaling of the input (raw) signal.

Due to the high frequency content of the filtered signal, the threshold based algorithm of stage 1 was not suitable to determine the time parameters. As a consequence, a slope-based algorithm (see Figure 6b) was selected, where the estimation of the entry and exit points were obtained by intersecting the threshold level with the slope lines of the rising and falling edges of each peak.

#### 4.1.3. Stage 3: Calibration and Correction Factor

At the final stage, a correction factor was added in order to compensate for systematic errors along the measurement chain. The main error contribution came from the microphone due to its slow response time at the exit of the cutting edge. The experimental procedure to determine the correction factors is described in Section 5.2.

### 4.2. Experimental Setup

Experiments of an end-milling process were carried out in a DMG 1035 three-axis machining center. An 8 mm diameter three-flute cutter rotating at 1200 rpm was used to machine a 7075 aluminum workpiece. The cutting forces were measured on a Kistler 9257A dynamometric platform (see Table 2) connected to a Kistler 5070 amplifier unit. In addition, a Knowles SPU1410LR5H-QB non-contact MEMS microphone (see Table 1) and a custom made conditioning board (see Figure 4) were used in order to measure the airborne acoustic emissions. In all tests, the microphone was placed at a distance of 500 mm perpendicular to the center of the workpiece.

**Figure 7:**
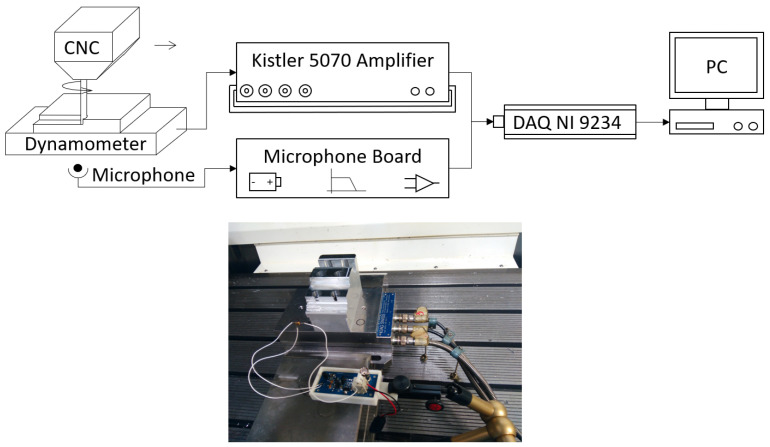
Scheme of the setup (**top**) and image of the sensors and the piece being machined (**bottom**).

The data acquisition of both signals was performed by a NI-9234 module for dynamic signals attached to a personal computer. The sampling rate was 51.2 kHz configured for bipolar input with a dynamic range of 10 V and a resolution of 24 bits. It should be noted that this device had a crosstalking specification of −110 dB, which was especially suitable considering the difference in magnitude of the signals that were compared. This setup is represented schematically in Figure 7.

## 5. Results and Discussion

In this work, two sets of experiments were conducted. First, a comparison between the cutting forces and the acoustic emission signals. This can be considered a qualitative waveform analysis to validate the accuracy of the method in the detection of the time parameters fed into the geometric model. Then, the performance of the prototype measurement system was tested under different machining conditions during end-milling operations.

### 5.1. Qualitative Waveform Analysis

The cutting forces are considered a good descriptor of the physics behind machining processes. The latter remains valid in the case of the end-milling process, and therefore, this knowledge can be used to validate the predictions of the time parameters obtained from the acoustic emission signal.

**Figure 8:**
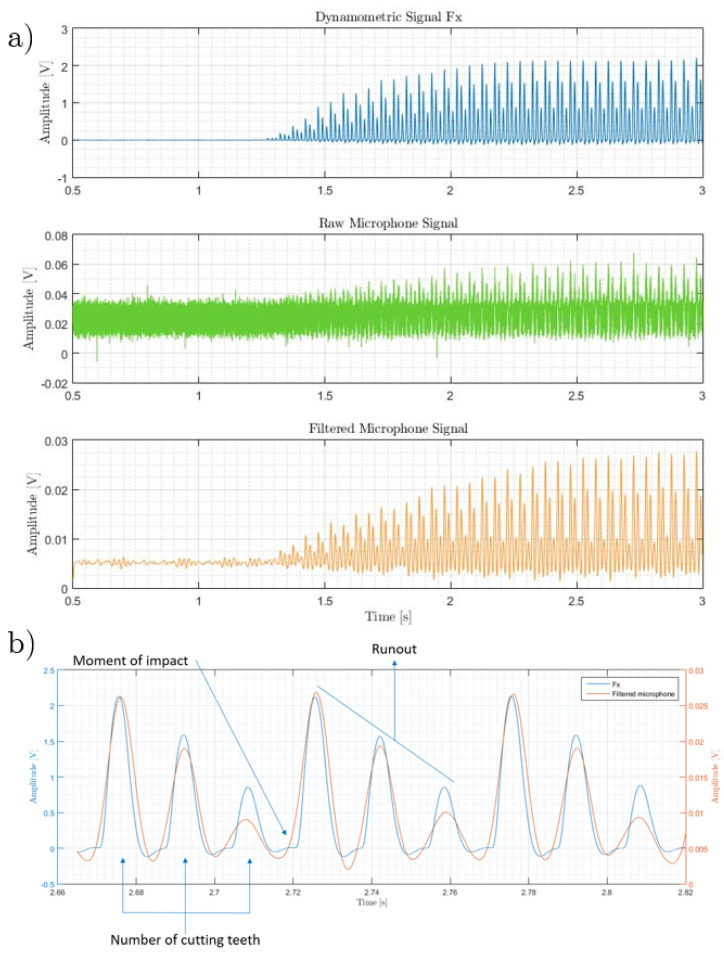
Experimental signals under analysis. (**a**) Cutting force signal (top), raw AE signal (middle), and filtered AE signal (bottom). (**b**) Zoomed section of the cutting force and filtered AE signals within the zone of uniform cut.

In Figure 8a three different waveforms are presented, the cutting force signal, the raw AE signal and the filtered AE signal, respectively. Although the amplitude of the background noise in the raw AE signal hid most of the features of the process, the filtered AE signal resembled the pattern of the cutting force. Figure 8b shows a zoomed section of the cutting force and the filtered AE signals within the zone of uniform cut. This reveals that both signals captured in amplitude and time the most important attributes of the process, such as the entrance and exit of the three cutting edges on each revolution of the spindle. Additionally, one can notice that these three peaks had different amplitudes, which could be an indication of tool runout [58].

### 5.2. Performance of the Prototype Measurement System

After the previous validation stage, the performance of the prototype measurement system was tested under different machining conditions during end-milling operations as shown in Table 3. In all runs, only the axial depth of cut was changed from 2 to 10 mm, increasing this value in steps of 2 mm. An additional test was carried out, in order to evaluate the response to sudden changes by increasing the axial depth of cut from 2 to 10 mm in a single step.

The mean value and standard deviation of the estimated depth of cut are presented in Table 4. These results were obtained from the analysis of signals within the zone of uniform cut, with a period of 7 s, equivalent to 140 revolutions of the cutter. In most cases, the error of the estimated depth of cut was larger than the nominal value. However, the standard deviation was consistently independent of the size of depth of cut increment between two different runs.

**Figure 9:**
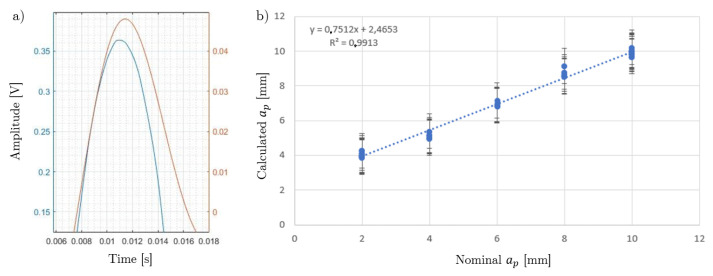
Experimental results. (**a**) Exit time delay between the cutting force signal (blue) and filtered AE signal (orange). (**b**) Calibration curve: estimated vs. nominal depth of cut.

The presence of large errors was a direct consequence of the slow time response of the microphone, which gave a poor estimation of the exit time of the cutting edge (see Figure 9a). However, the exit time difference between the cutting force and the filtered AE signal remained relatively constant and therefore, the predicted value can be adjusted in terms of a correction factor obtained from a calibration curve as shown in Figure 9b. This calibration curve was valid for the test conditions listed in Table 3. Any new set of machining conditions requires its own calibration process, which is the main drawback of this methodology.

On the other hand, the use of an adaptive filter and the geometric model of the end-milling process offers the potential to estimate the depth of cut even if the depth of cut varies during the cutting operation. However, this property requires further study and testing.

### 5.3. Precision of the Proposed Method

The experimental results after the calibration stage are shown in Table 5. The corrected estimates of the depth of cut, obtained from the filtered AE signal, are of the same order to the reference values given by the cutting forces. The absolute difference between these estimates is slightly above 10%, which supports the feasibility of this prototype system as an alternative to the traditional methods with the advantage of reduced cost and low invasiveness.

Furthermore, it should be noted that this algorithm is capable of working completely independent of the CNC control. If the nominal value of the depth of cut is provided as an input to the system, the difference between the estimates of the filtered AE signal and the values of cutting force drops to less than 1%, as can be seen in Table 6.

**Figure 10:**
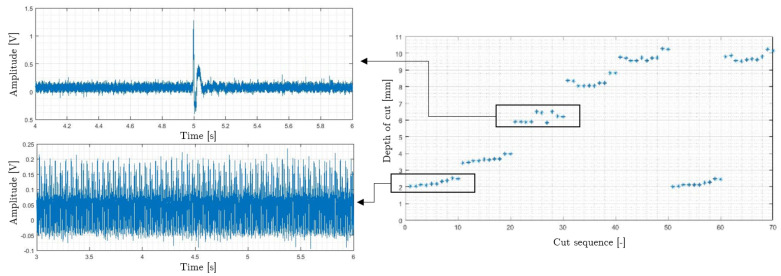
Effect of impacts and high level of background noise on the experimental results.

The applicability of this method relies on controlling the presence of any external disturbance, and further analysis should be performed. As an example, Figure 10 shows the calculated depth of cut values for all the test runs listed in Table 3. Note that the effect of higher background noise level and impacts can be seen for a_p_ = 2 mm and a_p_ = 6 mm, respectively.

## 6. Conclusions

The present contribution discusses a prototype measurement system with the ability to estimate the axial depth of cut during end-milling operations. This is performed by the analysis of airborne acoustic emissions registered by a non-contact MEMS microphone. The advantage of this system is the reduced cost and low invasiveness, which might be considered as an alternative to traditional methods with contact-based transducers mounted rigidly to the workpiece.

Here, a geometric model of the machining process, an adaptive filter and a calibration stage has been used to estimate the depth of cut during end-milling operations. The feasibility of this method has been tested experimentally in a CNC machining center under laboratory conditions. The estimations of the depth of cut are compared with the reference values given by the cutting forces. These errors are slightly above 10% when the algorithm is working completely independent from the CNC control, and it drops to less than 1% if the nominal depth of cut is fed into the algorithm.

Extending this methodology to industrial applications requires further study. For instance, the accuracy of the predicted values should be assessed in terms of the background noise level of the input signal. Moreover, the calibration process and the analysis of overlapping in multi-flute cutters should also be considered in future work.

## Figures and Tables

**Table 1:** Technical specifications of the SPU1410LR5H-QB microphone.

Range	10 Hz–10 kHz
Directivity	Omnidirectional
Sensitivity	−38 dBV/Pa
Signal to Noise Ratio	63 dB(A)
DC Output at $V_{dd}$ = 1.5 V	0.73 V
Total Harmonic Distortion at 1 kHz	0.15%

**Table 2:** Main characteristics of the dynamometer Kistler 9257A.

Dynamic range	±5 kN
Threshold	<0.01 N
Pretensioning direction	Vertical
Frequency range	1 Hz–2 kHz
Linearity, all ranges	<±1%

**Table 3:** Machining conditions of the experiment.

Depth of cut	a_p_ [mm]	2–10
Width of cut	a_e_ [mm]	1
Feed per tooth	f_z_ [mm]	0.08
Spindle speed	n [rpm]	1200
Tool flute number	N [-]	1
Tool diameter	D [mm]	8
Tool helix angle	λ_s_ [°]	30

**Table 4:** Experimental results for the estimated axial depth of cut.

Nominal a_p_ [mm]	Calculated a_p_ [mm]	STD [μm]	Error [mm]
2	4.14	174	2.14
4	5.21	185	1.21
6	7.10	280	1.10
8	8.70	300	0.70
10	9.80	259	−0.20
2	4.12	159	2.12
10	9.82	241	−0.18

**Table 5:** Comparison between the dynamometer and the microphone values.

	Dynamometer		Microphone		
Nominal a_p_ [mm]	Calculated a_p_ [mm]	STD [μm]	Calculated a_p_ [mm]	STD [μm]	Difference [%]
2	2.01	8	2.23	174	9.87
4	4.03	14	3.65	185	−10.41
6	5.99	23	6.17	280	2.92
8	8.04	31	8.30	300	3.13
10	10.30	39	9.76	259	−5.13

**Table 6:** Results with known nominal depth of cut.

	Dynamometer		Microphone		
Nominal a_p_ [mm]	Calculated a_p_ [mm]	STD [μm]	Calculated a_p_ [mm]	STD [μm]	Difference [%]
2	2.01	8	2.01	14	0.50
4	4.03	14	4.00	25	<0.01
6	5.99	23	5.99	38	−0.17
8	8.04	31	8.00	62	<0.01
10	10.30	39	10.01	53	0.10

## Abbreviations

The following abbreviations are used in this manuscript:

AE	Acoustic Emission
a_e_	Radial depth of cut, mm
*a_e_i__*	Axial depth of cut, mm
*a_p_i__*	Axial depth of cut of the flute i, mm
*a_p_a__*	Actual axial depth of cut, mm
φ*_en_*	Entry angle, rad
φ*_en_i__*	Entry angle of flute i, rad
φ*_pr_*	Projected angle of the cutting edge, rad
F_x_	Cutting force in X direction, N
T	Period of spindle rotation, s
D	Tool diameter, mm
f_z_	Feed per tooth, mm
n	Spindle speed, rpm
N	Tool flute number, -
λ_s_	Tool helix angle, rad
